# A panel of human neutralizing mAbs targeting SARS-CoV-2 spike at multiple epitopes

**DOI:** 10.1038/s41467-020-18159-4

**Published:** 2020-08-27

**Authors:** Tal Noy-Porat, Efi Makdasi, Ron Alcalay, Adva Mechaly, Yinon Levy, Adi Bercovich-Kinori, Ayelet Zauberman, Hadas Tamir, Yfat Yahalom-Ronen, Ma’ayan Israeli, Eyal Epstein, Hagit Achdout, Sharon Melamed, Theodor Chitlaru, Shay Weiss, Eldar Peretz, Osnat Rosen, Nir Paran, Shmuel Yitzhaki, Shmuel C. Shapira, Tomer Israely, Ohad Mazor, Ronit Rosenfeld

**Affiliations:** Israel Institute for Biological Research, Ness-Ziona, Israel

**Keywords:** Immunotherapy, Viral infection

## Abstract

The novel highly transmissible human coronavirus SARS-CoV-2 is the causative agent of the COVID-19 pandemic. Thus far, there is no approved therapeutic drug specifically targeting this emerging virus. Here we report the isolation and characterization of a panel of human neutralizing monoclonal antibodies targeting the SARS-CoV-2 receptor binding domain (RBD). These antibodies were selected from a phage display library constructed using peripheral circulatory lymphocytes collected from patients at the acute phase of the disease. These neutralizing antibodies are shown to recognize distinct epitopes on the viral spike RBD. A subset of the antibodies exert their inhibitory activity by abrogating binding of the RBD to the human ACE2 receptor. The human monoclonal antibodies described here represent a promising basis for the design of efficient combined post-exposure therapy for SARS-CoV-2 infection.

## Abstract (web summary)

Here, Noy-Porat, Makdasi et al. report the isolation of a panel of neutralizing mAbs selected against SARS-CoV-2 receptor-binding domain (RBD) from a phage display library constructed based on patient samples collected in the acute phase of the disease, which show efficient neutralizing activities against authentic virus in vitro.

## Introduction

The present global pandemic of coronavirus induced disease 19 (COVID-19), declared by the World Health Organization (WHO) as a public health emergency of international concern, is caused by the highly transmissible severe acute respiratory syndrome coronavirus 2 (SARS-CoV-2). To this date (end of June, 2020), about 10 million confirmed cases and 500,000 deaths have been reported worldwide^1^. Yet, there is no approved therapeutic drug specifically targeting the SARS-CoV-2. The novel coronavirus SARS-CoV-2, emerged as the seventh type of coronavirus infecting humans and the third most pathogenic preceded by the Severe Acute Respiratory Syndrome coronavirus (SARS-CoV) and the Middle East Respiratory Syndrome coronavirus (MERS-CoV).

Due to their exceptional antigen specificity, therapeutic monoclonal antibodies (mAbs) are considered an attractive candidate to target exposed antigenic sites on viruses, and prevent their infectivity^2^. While the therapeutic potential of mAbs, specifically targeting surface viral proteins was demonstrated for SARS-CoV^3^ and MERS^4,5^, only antibodies specific for Ebola are currently undergoing the more advanced clinical studies^6^.

SARS-CoV-2 utilizes the surface homotrimeric spike glycoprotein (S) as a major mediator of cellular infection^7,8^. The SARS-CoV-2 S protein is composed of two distinct subunits, S1 and S2. The S1 subunit contains the receptor-binding domain (RBD), known to bind the Angiotensin-Converting Enzyme 2 (ACE2) receptor on host cell surfaces and the S2 subunit mediates the fusion of the viral and cellular membranes, essential for viral entry into the cell^8,9^. The receptor interaction site on S1 is considered the main target for efficient neutralization of cell infection and therefore a prime candidate for therapeutic antibody development^10–14^. Although the S protein of the SARS-CoV and SARS-CoV-2 share 77.5% identity, most of the mAbs isolated against SARS-CoV reportedly failed to cross-neutralize the SARS-CoV-2 virus^15–17^.

For designing optimal therapeutic strategies, there is an urgent need for the identification of neutralizing monoclonal antibodies that specifically target SARS-CoV-2. Such antibodies may be identified either in patients in the course of illness/recovery, or in immunized animals. In addition, alternative antibody discovery strategies may be applied, e.g., using synthetic Ab libraries. Recently, first two mAbs, elicited against the SARS-CoV-2, were reported^18^. Furthermore, efficient post-exposure therapy in humans, may require integration of several noncompeting mAbs, ideally neutralizing the virus infectivity by different mechanisms. Such combined therapies are expected to provide superior control of potential neutralizing escape variants^11,19^.

Here we describe the isolation of a panel of neutralizing mAbs selected against the SARS-CoV-2 RBD from phage display library constructed based on patient samples collected in the acute phase of the disease. These specific antibodies were found to recognize distinct epitopes and can potentially be used either for therapy or immune prophylaxis.

## Results and discussion

### Isolation of anti-SARS-CoV-2 antibodies

**Fig. 1:**
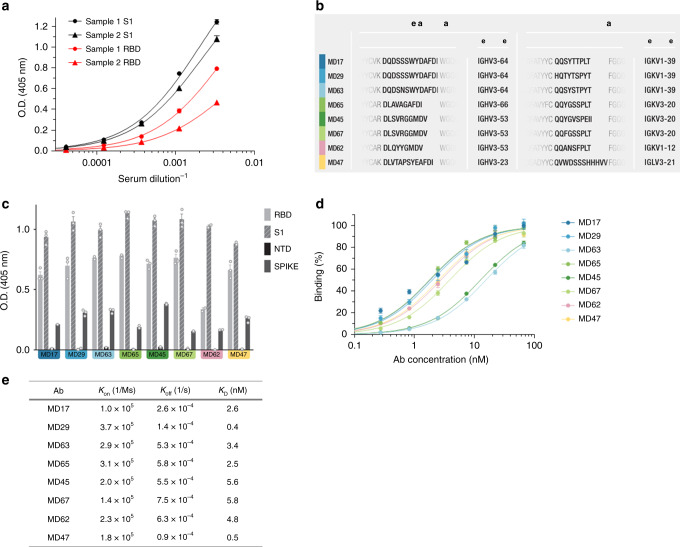
Characterization of the novel human anti-SARS-CoV-2 antibodies. **a** Binding curves of polyclonal antibodies in serially diluted serum samples (*n* = 3 wells per dilution) of COVID-19 patients obtained by ELISA using S1 or RBD as coating antigen. Data represent average of triplicates ±SEM. **b** Amino acid sequences of the HCDR3 and LCDR3 of the selected antibodies and their respective germ line genes. **c** Specificity of the selected antibodies determined by ELISA (*n* = 3 wells per sample) against the indicated SARS-CoV-2 proteins. Data represent average of triplicates ±SEM. **d** Reactivity profile of antibodies determined by ELISA (*n* = 3 wells per dilution), using S1 as the coating antigen. Data is presented as binding percent of B_max_ for each antibody. The values represent average of triplicates ±SEM. **e** Binding characteristics of the monoclonal antibodies determined using biolayer interferometry. All antibodies were biotinylated, immobilized to the sensor and interacted with increasing amounts of RBD. Binding kinetics were fitted using the 1:1 binding model.

Several blood samples, either from COVID-19 convalescence or from patients with severe ongoing disease, were evaluated for titers of RBD binding and viral neutralization activity. Two blood samples derived from patients at the acute phase of disease, exhibiting the highest neutralizing ability (NT_50_ > 5000) and demonstrating significant binding to both S1 subunit [DIL_50_ (half-dilution value) of 494 and 473] and RBD (DIL_50_ of 252 and 226; Fig. 1a), were subsequently selected for antibody library generation.

A phage display (PD) single-chain Fv (scFv) library, representing approx. 10 million distinct antibodies, was constructed. With the objective of isolating neutralizing Abs, three consecutive enrichment steps of panning were performed against both mFc-S1 and huFc-RBD. Clones resulting from these enrichment steps, were tested by ELISA for their ability to bind huFc-RBD or huFc-NTD. RBD-specific binders resulting from this screen were expressed as full-length antibodies (in a scFv-Fc format) for further analysis. Subsequently, eight RBD-specific antibodies carrying unique sequences were selected (Fig. 1b; Supplementary Fig. 1). Of note, no clones exhibiting anti NTD specificity were detected by the ELISA of those resulting from enrichment of the library against the S1 subunit. Furthermore, the clones resulting from enrichment using the full-length S1 subunit, were identical to those obtained following enrichment against RBD only.

Binding assays of these eight antibodies confirmed their specificity to the spike protein, the S1 subunit, and the RBD, while no reactivity was observed against the spike N terminal domain (NTD) of the spike protein (Fig. 1c). Evaluation of the Abs affinity toward S1 by ELISA evidenced apparent K_D_ values of 1.8–3.8 nM of six of the antibodies, and a K_D_ values of 12.7 and 15.6 nM of MD45 and MD63, respectively (Fig. 1d). To further characterize these antibodies, biolayer interferometry (BLI) measurements of the antibodies affinity specifically toward RBD was conducted, revealing K_D_ values of 0.4–5.8 nM for all antibodies, with MD29 showing the highest affinity and MD67 showing the lowest affinity (Fig. 1e; Supplementary Fig. 2).

Additional sequence analysis by IgBlast^20^ (Fig. 1b) revealed that antibodies MD17, MD29, and MD63 share common germ line origin of both their VH and VK (IGHV3-64 and IGKV1-39, respectively). Similarly, MD45 and MD67, share common VH and VK germ lines (IGHV3-53 and IGKV3-20). The VH of MD62, is similar to the one of MD45 and MD67, accompanied by a unique VK (IGKV1-12), while MD65 shared the same VK with MD45 and MD67, accompanied by a unique VH (IGHV3-66). MD47 originated from unique VH (IGHV3-23) and was the only mAb, carrying VL (IGLV3-21).

### Classification of antibody epitopes

**Fig. 2:**
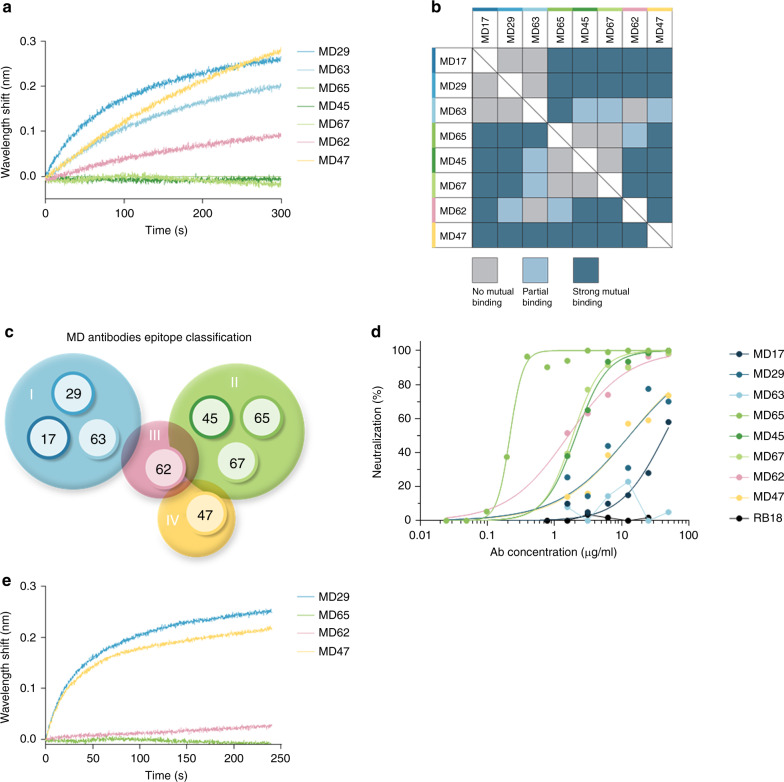
Epitope binning and SARS-CoV-2 neutralization. **a** Biolayer interferometry (BLI) was applied for the epitope binning experiments. Representative assay results are shown for MD65 mAb. The purified antibody was biotinylated, immobilized on streptavidin sensor and saturated with RBD. The complex was then incubated for 300 s with each one of the indicated antibodies. Time 0 represents the binding to the MD65-RBD complex. **b** Complete epitope binning of the eight selected MD monoclonal antibodies. Binding was evaluated by the ability of each pair of antibodies to simultaneously bind RBD, using biolayer interferometry. The matrix presents the concluded epitope specificity on the basis of the various competition experiments; see Supplementary Fig. 3 for the detailed competition profiles obtained by the binning experiments. **c** Four noncompeting RBD binding epitopes were identified and accordingly classified into four groups: I (blue), II (green), III (pink) and IV (yellow). **d** SARS-CoV-2 in vitro neutralization using plaque reduction neutralization test (PRNT). Neutralization potency was determined by the ability of each antibody (at indicated concentrations) to reduce plaques formation; results are expressed as percent inhibition of control without Ab. The values represent average of duplicates. **e** Binding of human ACE2 to RBD in the presence of neutralizing antibodies (representing each of the epitope groups) was tested by BLI. Each of the biotinylated antibodies was immobilized on streptavidin sensor, saturated with RBD, washed and incubated with recombinant human ACE2 for 300 s. Time 0 represents the binding of the ACE2 to the antibody-RBD complex.

SARS-CoV-2 spike RBD is known to mediate the binding of the human ACE2 receptor and thus, this domain is considered as main target for neutralizing mAbs. However, direct blocking of the RBD-ACE2 interaction is not the exclusive modality by which neutralizing antibodies can exert their effect^21^. Consequently, the selected mAbs, were classified on the basis of their epitope specificity determined by BLI epitope binning. In this assay, each individual antibody was biotinylated, immobilized to a streptavidin sensor, loaded with RBD and then challenged with each of the other antibodies. Simultaneous binding of the second antibody to RBD induces a wavelength shift in the interference pattern, which indicates that the two antibodies bind to non-overlapping epitopes^21^. Conversely, if the two antibodies bind the same or partially-overlapping epitope on RBD, no or very low wavelength shift, respectively, is induced. As a representative example, sensograms of the various antibody interactions with a pre-complexed MD65-RBD is shown (Fig. 2a). Antibody MD65 used as a negative control, and did not elicit any wavelength shift, as expected. In contrast, antibodies MD29, MD47, MD62, and MD63 induced a marked wavelength shift indicating that they could bind to RBD simultaneously with MD65. The analysis revealed that antibodies MD45 and MD67 could not bind to RBD in the presence of MD65, indicating that these three antibodies target the same epitope. Analysis was then performed for the next seven antibodies (Supplementary Figs. 3 and 4) and the ability of each pair to simultaneously bind RBD was determined (Fig. 2b). Results ranged from full to no competition and enabled the classification of the mAbs into four groups recognizing distinct epitopes (Fig. 2c): I (MD17, MD29, and MD63); II (MD45, MD65, and MD67); III (MD62) and IV (MD47). A lack of reciprocality in the binning experiments was observed in the case of antibodies MD45 and MD47. These antibodies showed strong mutual binding in one experimental setup (MD45-bound sensor vs MD47 as competitor), while in the reverse experimental setup (MD47-bound sensor vs MD45 as competitor) the antibody MD45 failed to reveal any binding to RBD (Supplementary Fig. 3). This phenomenon is frequent in the case of antibody pairs exhibiting significant differences of affinity to their respective epitopes. Antibodies MD45 and MD47 indeed exhibit different affinity values (of 10 fold), as shown in the table in Fig. 1e. This lack of reciprocality in experimental evaluation by epitope binning was previously reported^22–24^.

Most notably, the classification of the antibodies on the basis of their specific targeted antigenic epitopes, is strongly supported by their observed sequence similarity, as discussed above. Group I mAbs shared the same V germ lines and were found to target the same epitope. Similarly, mAbs MD45 and MD67 which shared the same V germ lines and the same epitope, were classified as group II. Although MD65 differ in its VH germ line, he was included in this group as well. On the other hand, MD62 sharing the same VH germ line with MD45 and MD67, appears to bind a distinct epitope (III). Finally, MD47 exhibited both a unique sequence and targeted a unique epitope (Fig. 2c).

Recently, the RBD-located epitope recognized by the SARS-CoV specific antibody CR3022, was determined^15,17^. We chose to use this antibody in order to further determine the epitopes recognized by the antibodies described in this report. Therefore, a recombinant in-house version of this antibody was generated (Supplementary Data) and used in epitope binning assays, together with the selected set of the novel mAbs. CR3022 IgG was immobilized to the BLI sensor, loaded with RBD and further challenged with each of the selected mAbs. Group I mAbs were found to compete with the CR3022 as evidenced by the lack of interaction with the mAb-RBD complex (Supplementary Fig. 5). The remaining five tested mAbs did bind the RBD in the presence of the CR3022 antibody. These results suggest that Group I antibodies bind to the RBD epitope that spans the RBD residues 369–386, previously defined as the CR3022 epitope^17^.

In a previous study, a human anti-SARS-CoV-2 spike antibody (named S309) was shown to recognize a proteoglycan epitope^25^. This report prompted us to interrogate whether the ability of the novel antibodies described here to recognize and bind RBD also involves glycosylated moieties. To this objective, the binding ability of representative antibodies from each epitope-group was tested using an *E.coli*-expressed non-glycosylated recombinant version of RBD. The data demonstrate that glycosylation is not essential for recognition of RBD (Supplementary Fig. 6).

### SARS-CoV-2 neutralization by the novel human antibodies

The neutralization potency of the antibodies was evaluated by plaque reduction neutralization test (PRNT) using VeroE6 cells infected with the pathogenic SARS-CoV-2. A fixed amount of the virus was incubated with increasing concentrations of each antibody, the mixture was then added to the cells, and the number of plaques was quantified 48 h later. Antibodies MD45, MD67, MD62, and MD65 displayed the highest neutralization potency, with a neutralization dose needed to inhibit 50% of the plaques (NT_50_) of 2.1, 1.9, 1.6, and 0.22 μg/ml, respectively. MD65 exhibited the highest neutralization capacity amongst the entire set of antibodies (Fig. 2d). Interestingly, antibodies MD17, MD29, and MD63 shared DNA sequence homology indicative of a common germ line and competed for the same epitope, yet only the first two showed neutralizing activity (NT_50_ of 43 and 13 μg/ml, respectively). This discrepancy may be explained by differences in the affinities of the antibodies in this group. A recent report similarly documented that antibody CR3022 (targeting an epitope overlapping with MD17 and MD29, see above) does not neutralize the novel SARS-CoV-2 presumably due to its low binding affinity to the RBD (115 nM)^17^. Additional antibody tested in this study, MD47, which represents a unique epitopic group, also showed neutralizing activity with NT_50_ of 13 μg/ml. Note that irrelevant Ab, RB18 (scFv-Fc) targeting abrin toxin, showed no neutralization against the virus.

One of the major mechanisms of antibody-mediated neutralization of SARS-CoV-1 and CoV-2 involves abrogation of the interaction between the viral spike protein and the human ACE2 receptor^26^. Therefore, in the current study, this aspect was further assessed by addressing the ability of representative antibodies from each epitope-group to prevent the binding of RBD to the huACE2 receptor by BLI analyses. Each antibody was immobilized on a BLI sensor, reacted with RBD and subsequently the ability of recombinant huACE2 to bind to the Ab-RBD complex was determined. The analysis established that MD29 and MD47 did not interfere with the binding of RBD to its receptor (Fig. 2e). This observation is in agreement with a previous report showing that antibody CR3022 (which recognizes the same epitope as antibody MD29) does not inhibit the RBD-ACE2 interaction in spite its ability to bind and neutralize SARS-CoV^17^. As opposed to MD29 and MD47, antibodies MD62 and MD65 completely inhibited the binding of RBD to ACE2. It is interesting to note that these two antibodies exhibited the highest neutralization potency (Fig. 2d), substantiating the essentiality of RBD-ACE2 prevention for efficient inhibition of viral activity.

To conclude, we report the isolation and characterization of a set of fully human, SARS-CoV-2 neutralizing antibodies that target four distinct epitopes on the spike RBD. As neutralizing antibodies are generally known to be useful as post-exposure therapy for viral infection and more specifically for treatment of human corona viral diseases^3,27^, we suggest that these antibodies might serve as an efficient treatment of COVID-19 patients or for prophylaxis immunization. Furthermore, since these neutralizing antibodies target different epitopes, they can be combined to further improve treatment efficacy and to reduce the risk of the emergence of treatment-escaping viral variants.

## Methods

### Blood samples

Sera and whole blood samples collected from five convalescent or severe COVID-19 patients were obtained under written inform consent and treated in accordance with the biosafety guidelines of the IIBR in BL3 facility. PBMCs were separated from fresh whole blood using density centrifugation by Ficoll. Sera samples were heat-inactivated (20 min at 60 °C) prior to use for binding or neutralizing assays. The study was approved by the Sheba Medical Center IRB Ethical Committee as well as by the Baruch Padeh Medical Center IRB Ethical Committee.

### Phage display scFv libraries construction and phage Abs selection

Total RNA was purified from PBMCs using RNeasy mini kit (Qiagen GmbH, Germany). CDNA synthesis was performed using Verso cDNA synthesis kit (Thermoscientific, USA) and used as a template for Abs variable region coding fragments amplification, as detailed in the Supplementary Data. Briefly, heavy and light Ig variable domains (VH and VL) were amplified, using specific primer set (Supplementary Table 1). The VH and VL used in PCR overlap extension reaction, resulted in scFv repertoire cloned into pCC16 phagemid vector^28^ using *Nco*I/*Not*I. Total of 9.2 × 10^6^ independent clones obtained, representing the library complexity.

For phage production, 25 ml of logarithmic bacteria culture (OD_600_ = 0.6) in YPD supplemented with 100 µg/ml ampicillin and 1% glucose (YPD-Amp-Glu) were infected with M13KO7 helper phage (New England Biolabs, USA) at 7 × 10^9^ plaque-forming unit (PFU) per ml (~1:20 multiplicity of infection) by incubating at 37 °C for 30 min without shaking, followed by 30 min at 120 rpm. Infected cells were harvested by centrifugation (1800 × *g* for 5 min) and resuspended in 100 ml YPD supplemented with 100 µg/ml ampicillin and 50 µg/ml kanamycin. After overnight growth at 30 °C at 200 rpm, the cells were removed by centrifugation (1800 × *g* at 4 °C for 10 min). The culture supernatant containing the phages was filtered through a 0.45 µm filter and then precipitated with 1/5 volume of 20% PEG6000 (polyethylene glycol) in a 2.5 M NaCl solution, for 2 h on ice. Phage particles were pelleted by centrifugation (9000 *×* *g* at 4 °C for 1 h) and re-dissolved in 5 ml Dulbecco’s Phosphate Buffered Saline (PBS; Biological Industries, Israel).

Panning was performed against the recombinant huFc-RBD (prepared as described below) and mFc-S1 (Sino Biological Inc., USA) directly absorbed to polystyrene plates (Maxisorb 96-well microtiter plates; Nunc, Sigma-Aldrich, USA) and against biotinylated-huFc-RBD (biotinylation performed using a commercial kit: EZ-Link sulfo-NHS-biotin, Pierce-Thermoscientific, USA) attached to streptavidin-coated magnetic beads (Dynabeads; Invitrogen, USA). All routine phage display techniques were performed essentially as described^29^. Blocking of plates, beads and phages was conducted for 60 min using two blocking solutions: 3% BSA (in PBS) or 2% skimmed milk and 0.05% Tween20 in PBS. The blocking solutions were alternated between panning cycles. All washing steps were performed using PBST (PBS containing 0.05% Tween20 and 2% BSA) or PBS. For each panning cycle, 5 µg/ml antigen was used to coat the polystyrene plate and after an overnight incubation, plates were washed and blocked. 20 µg biotinylated-huFc-RBD were incubated with 100 µl streptavidin-coated magnetic beads for 30 min, followed by blocking. For the first panning cycle, ~1 × 10^11^ phages were incubated with the antigen-coated plates for 60 min or with the blocked beads for 90 min, followed by a total of three washes with PBST for the plates and four washing steps (x2 with PBST and x2 with PBS) for the beads. Phages were eluted by incubation with 1 ml of 100 mM Triethylamine (Sigma, Israel) for 30 min and following neutralization (in 200 µl 1 M Tris-HCl, pH 7.4), were used to infect 5 ml of *E.coli* TG1 strain, by incubation at 37 °C for 30 min without shaking followed by 30 min at 120 rpm. The bacterial culture was plated on YPD-Amp-Glu agar, and incubated overnight at 30 °C. Clones were harvested into 5 ml YPD-Amp-Glu with 20% glycerol solution and phage production for the next round of panning was conducted in 10 ml medium, as described above. Two additional panning cycles were conducted essentially similarly, with the following modifications: 10^10^ and 10^9^ phages were used as input (for the 2nd and 3rd cycles, respectively) and the washing steps were increased to include six washes of PBST for the antigen-coated plates and 10 washes (x8 PBST and x2 PBS) for the beads.

Single colonies were randomly picked from the third cycle output and screen of specific binders was performed, using phage ELISA against huFc-RBD Vs huFc-NTD.

### Single-chain Fv (scFv) individual clone diversity and sequence verification

TAB-RI_For (CCATGATTACGCCAAGCTTTGGAGCC) and CBD-AS_Rev (GAATTCAACCTTCAAATTGCC) phagemid specific primers were used for colony PCR and sequence analysis of scFv Ab individual clones (Supplementary Fig. 1). Colony PCR products, were analyzed on 1.5% agarose gel, to confirm the intact of the scFv. Restriction fragment size polymorphism (RFLP), was performed using MvaI (FastDigest #FD0554; Thermoscientific, USA) to evaluate sequence variability of scFv individual clones. Following colony PCR, 5 µl of the PCR products were taken directly for restriction with 0.5 µl MvaI and 1 µl buffer x10 (provided by the manufacturer) in a 10 µl reaction volume. Restriction was conducted for 1 h at 37 °C, and the entire reaction mix was then resolved on 3% agarose gel. Nucleic acid sequence analysis of individual scFv fragments, was performed to the colony PCR product, using SeqStudio Genetic Analyzer (Applied Biosystems, USA).

### Expression of SARS-CoV-2 spike recombinant protein

Mammalian cell codon-optimized nucleic sequence, coding for SARS-CoV-2 spike glycoprotein (GenPept: QHD43416 ORF [https://www.ncbi.nlm.nih.gov/protein/1791269090]), was used to design pcDNA3.1+ based expression plasmids, mediating recombinant expression of the entire spike glycoprotein (amino acids 1–1207), receptor-binding domain (RBD; amino acids 1–15 and 318–542), N-terminal domain (NTD; amino acids 1–305) and S1 (amino acids 1–685). A stabilized soluble version of the spike protein was designed by inclusion of the proline substitutions at positions 986 and 987, and disruptive replacement of the furin cleavage site RRAR (residues at position 682-685) with GSAS, as reported^30,31^. C-terminal his-tag as well as streptag, was included in all constructs in order to facilitate protein purification. Expression of the recombinant proteins was performed using ExpiCHO^TM^ Expression system (Thermoscientific, USA) following purification using HisTrap^TM^ (GE Healthcare, UK) and Strep-Tactin^®^XT (IBA, Germany).

In addition, huFc-RBD fused protein was expressed using previously designed Fc-fused protein expression vector^32^, giving rise to a protein comprising of two RBD moieties (amino acids 318–542, see accession number of the S protein above) owing to the homodimeric human (gamma1)Fc domain (huFc). Expression of the recombinant proteins performed using ExpiCHO^TM^ Expression system (Thermoscientific, USA) following purification using HiTrap Protein-A column (GE healthcare, UK). All purified proteins preserved in PBS.

### Production of scFv-Fc antibodies

Phagemid DNA of the desired clones were isolated using QIAprep spin Miniprep kit (Qiagen, GmbH, Hilden, Germany), and the entire scFv was cloned into a pcDNA3.1+ based expression vector that was modified, providing the scFv with the human (IgG1) CH2-CH3 Fc fragments, resulting in scFv-Fc antibody format. ScFv-Fc were expressed using ExpiCHO^TM^ Expression system (Thermoscientific, USA, Cat# A29133) and purified on HiTrap Protein-A column (GE healthcare, UK). The integrity and purity of the scFv-Fc Abs were analyzed using SDS-PAGE (Supplementary Fig. 7).

### ELISA

Direct ELISA^33^ consisted of coating microtiter plates with 2 μg/ml of recombinant SARS-CoV-2 spike, S1 domain, RBD or NTD subunits. For phage ELISA, HRP-conjugated anti-M13 antibody (Sino Biological, USA, Cat# 11973-MM05T-H lot HO13AU601; used at 1:5000 working dilution) was used following detection with TMB substrate (Millipore, USA). ELISA of both sera and recombinant scFv-Fc human antibodies was applied with AP-conjugated Donkey anti-human IgG (Jackson ImmunoResearch, USA, Cat# 709-055-149 lot 130049; used at 1:2000 working dilution) following detection using PNPP substrate (Sigma, Israel).

### Biolayer interferometry for affinity measurements and epitope binning

Binding studies were carried out using the Octet system (ForteBio, USA, Version 8.1, 2015) that measures biolayer interferometry (BLI). All steps were performed at 30 °C with shaking at 1500 rpm in a black 96-well plate containing 200 μl solution in each well. Streptavidin-coated biosensors were loaded with biotinylated scFv-Fc antibody (1–5 µg/ml) to reach 0.7–1 nm wavelength shift followed by a wash. The sensors were then reacted for 300 s with increasing concentrations of monomeric RBD (association phase) and then transferred to buffer-containing wells for another 600 s (dissociation phase). Binding and dissociation were measured as changes over time in light interference after subtraction of parallel measurements from unloaded biosensors. Sensorgrams were fitted with a 1:1 binding model (Supplementary Fig. 2) using the Octet data analysis software 8.1 (Fortebio, USA, 2015), and the presented values are an average of several repeated measurements. For the binning experiments of antibodies pairs, antibody-loaded sensors were incubated with a fixed RBD concentration (300 nM), washed and incubated with the non-labeled antibody counterpart. In each set of experiments, the background signal was obtained from a parallel sensor incubated with the homologous antibody and sensograms are presented after subtraction of the background signal (Supplementary Fig. 3). In addition, binning experiments were performed in the absence of RBD, in order to validate the absence of non-specific binding (Supplementary Fig. 4). To study the binding of ACE2 to RBD in the presence of antibodies, antibody-loaded sensors were incubated with a RBD, washed and incubated with huACE (10 μg/ml; Sino Biological Inc., USA).

### Cells

Vero E6 (ATCC® CRL-1586^TM^) were obtained from the American Type Culture Collection (Summit Pharmaceuticals International, Japan). Cells were used and maintained in Dulbecco’s modified Eagle’s medium (DMEM) supplemented with 10% fetal bovine serum (FBS), MEM non-essential amino acids, 2 nM L-Glutamine, 100 Units/ml Penicilin, 0.1 mg/ml streptomycin and 12.5 Units/ml Nystatin (Biological Industries, Israel). Cells were cultured at 37 °C, 5% CO_2_ at 95% air atmosphere.

ExpiCHO-S (Thermoscientific, USA, Cat# A29127) were used for expression of recombinant proteins as described above.

### Plaque reduction neutralization test (PRNT)

SARS-CoV-2 (GISAID accession EPI_ISL_406862) was kindly provided by Bundeswehr Institute of Microbiology, Munich, Germany. Stocks were prepared by infection of Vero E6 cells for two days. When viral cytopathic effect (CPE) was observed, media were collected, clarified by centrifugation, aliquoted and stored at –80 °C. Titer of stock was determined by plaque assay using Vero E6 cells. Handling and working with SARS-CoV-2 was conducted in BL3 facility in accordance with the biosafety guidelines of the IIBR.

For plaque reduction neutralization test (PRNT)^34^, Vero E6 cells were seeded overnight (as detailed above) at a density of 0.5 × 10^6^ cells/well in 12-well plates. Antibody samples were 2-fold serially diluted (ranging from 100 to 0.1 μg/ml) in 400 μl of MEM supplemented with 2% FBS, MEM non-essential amino acids, 2 nM L-Glutamine, 100 Units/ml Penicilin, 0.1 mg/ml streptomycin and 12.5 Units/ml Nystatin (Biological Industries, Israel). 400 μl containing 300 PFU/ml of SARS-CoV-2 virus were then added to the mAb solution supplemented with 0.25% guinea pig complement sera (Sigma, Israel) and the mixture incubated at 37 °C, 5% CO_2_ for 1 h. Monolayers were then washed once with DMEM w/o FBS and 200 μl of each mAb-virus mixture was added in duplicates to the cells for 1 h. Virus mixture w/o mAb was used as control. 2 ml overlay [MEM containing 2% FBS and 0.4% tragacanth (Sigma, Israel)] were added to each well and plates were further incubated at 37 °C, 5% CO_2_ for 48 h. The number of plaques in each well was determined following media aspiration, cells fixation and staining with 1 ml of crystal violet (Biological Industries, Israel). NT_50_ was defined as mAb concentration at which the plaque number was reduced by 50%, compared to plaque number of the control (in the absence of Ab).

### Reporting summary

Further information on research design is available in the Nature Research Reporting Summary linked to this article.

## Supplementary information

Supplementary Information

Reporting Summary

## Data Availability

Antibodies are available (by contacting Ohad Mazor from the Israel Institute for Biological Research; ohadm@iibr.gov.il) for research purposes only under an MTA, which allows the use of the antibodies for non-commercial purposes but not their disclosure to third parties. All other data are available from the corresponding authors upon reasonable requests. Source data are provided with this paper.

## Source data

Source Data
