# Supplementary material for: A panel of human neutralizing mAbs targeting SARS-CoV-2 spike at multiple epitopes
Source: Nat Commun. 2020 Aug 27;11:4303. doi: 10.1038/s41467-020-18159-4 (PMC7452893; doi:10.1038/s41467-020-18159-4)
Supplement: Supplementary file 1 — Supplementary Information [file 41467_2020_18159_MOESM1_ESM.pdf]

Supplementary Information

A panel of human neutralizing mAbs targeting SARS-CoV-2 spike at multiple epitopes

Tal Noy-Porat *et al*

Supplementary data

CR3022 in house version preparation: Heavy and Kappa variable domain sequences (GenBank DQ168569.1 and DQ168570.1) were obtained from GenScript and cloned into pcDNA3.1+ expression vector, harboring human IgG1 Fc, to obtain full-length IgG antibody. The mAb integrity was confirmed by ELISA prior to use.

Phage-display library construction: Heavy and light Ig variable domains (VH and VL) amplification was performed directly from cDNA, using V/J specific set of primers (indicated by upper case in Table S1). Amplification performed separately for each primer pair (1 min 95°C; 30 cycles of: 20 s, 94°C; 30 s, 55°C; 1 min, 72°C; 5 min, 72°C). PCR products were directly used as a template for a second amplification (1 min 95°C; 20 cycles of: 20 s, 94°C; 30 s, 57°C; 1 min, 72°C; 5 min, 72°C), using the above listed primers, adding flanking sequences (indicated by lower case) introducing restriction recognition sites for subsequent cloning into pCC16 phagemid vector. Following gel purification of the variable fragments [using QIAquick Gel extraction kit (Qiage, Germany)] assembly PCR performed for 200 ng of VH and Vκ/Vλ products (in a 1:1 ratio). First 10 assembly cycles were performed without primers addition (95°C, 1 min; 10 cycles of: 95°C, 15 s; 60°C, 30 s; 72°C, 30 s; 72°C, 5 min). Next, products were diluted 1:10 in fresh reaction mixture including the ASS1 primer pair for an additional 30 cycles (95°C, 1 min; 30 cycles of: 95°C, 15 s; 65°C, 20 s; 72°C, 1 min; 72°C, 5 min).

For PD library construction, pCC16 plasmid and assembled scFvs were digested with 5 U each of NcoI and NotI restriction enzymes (FastDigest, Thermo Scientific, USA), in a 50 µL reaction volume, 2 hrs. at 37°C. The resulted pCC16 vector was purified from 1% agarose gel using the QIAquick Gel extraction kit, and the resulted scFv inserts were purified using the QIAquick PCR purification kit (Qiagen, Germany). The scFv was than cloned into 200 ng of the pCC16 vector (5:1 ratio; insert: vector), in a 15 µL reaction volume, using 6 U of ligase (T4 HC ligase; Thermo Scientific, USA), with 2 hr incubation at RT and further overnight incubation at 4°C, followed by inactivation at 70°C for 5 min. Each ligation reaction was transformed to 30 µL of E. coli TG1 electrocompetent cells (Lucigen, USA), and a total of 48 cloning reactions were performed. The transformed bacteria, containing the final scFv libraries, were plated on YPD agar (BD, USA) supplemented with 100 µg/mL ampicillin and 100 mM glucose and, after an overnight culture at 30°C, were harvested, aliquoted and stored at -80°C.

Supplementary Figures

Supplementary Figure 1

Heavy chain

MD17- EVQLLESGGGLVQPGGSLRLSCSASGFTFSTYVMNWVRQAPGKGLEHVSGISSDGGITYYADSVKGRFTISRDNSKNTLYLQMSSLRVEDTAVYYCVKDQDSSSWYDAFDIWGQGTMVTVSS

MD29- QMQLVQSGGGLVQPGGSLRLSCSASGFTFSTYVMNWVRQAPGKGPEHVSGISSDGGITYYADSVKGRFTISRDNSKNTLYLQMSSLRVEDTAVYYCVKDQDSSSWYDAFDIWGQGTMVTVSS

MD63- QVTLKESGGGVVQPGRSLRLSCAASGFTFSSYAMHWVRQAPGKGLEHVSAISSDGGITYFADSVKGRFTISRDNSRNTLFLQMSSLRVEDTAVYYCVKDQDSNSWYDAFDIWGRGTMVTVSS

MD65- EVQLVESGGGLVQPGGSLGLSCAASGVTVSSNYMNWVRQTPGKGLEWVSVIYSGGSTYYADSVKGRFTISRDNSKNTLYLQMNSLRAEDTAVYYCARDLAVAGAFDIWGQGTMVTVSS

MD45- EVQLVQSGGGLVQPGGSLRLSCAASGFTVSSNYMTWVRQAPGKGLEWVSVIYSGGSTFYADSVKGRFTISRHNSKNTLYLQMNSLRAEDTAVYYCARDLSVRGGMDVWGQGTMVTVSS

MD67- EVQLVESGGGLVQPGGSLRLSCAASGFTVSSNYMTWVRQAPGKGLEWVSVIYSGGSTFYADSVKGRFTISRHNSKNTLYLQMNSLRAEDTAVYYCARDLSVRGGMDVWGQGTTVTVSS

MD62- EVQLVQSGGGLIQPGGSLRLSCAASGVTVSSNYMTWVRQAPGKGLEWVSLIYSGGSTYYADSVKGRFTISRDNSKNTLYLQMNSLRAEDTAVYYCARDLQYYGMDVWGQGTMVTVSS

MD47- QMQLVQSGGGLVQPGGSLRLSCAASGFTFSSYAMSWVRQAPGKGLEWVSAISSGGSTYYADSVKGRFTISRDNSKNTLYLQMNSLRAEDTAVYYCAKDLVTAPSYEAFDIWGQGTMITVSS

Light chain

MD17- DIVMTQSPSSLSASVGDRVTITCRASQSISNYLNWYQQKLGKAPKLLTYAASRLQSGVPSRFSASGSGTEFTLTISSLQPEDFATYYCQQSYTTPLTFGGGTKLEIK

MD29- NIRLTQSPSSLSASVGDRVTITCRATQSISSYLNWYQQKPGKAPNLLIYAASSLQSGVPSRFSGSGSGTDFTLTISSLQPEDFATYYCHQTYTSPYTFGQGTKLEIK

MD63- VIWLTQSPSSLSASVGDRVTITCRASQSISSYLNWYQQKPGKAPKLLIYAASSLQSGVPSRFSGSGSGTDFTLTISSLQPEDFATYYCQQSYSTPYTFGQGTKLEIK

MD65- EIVMTQSPGTLSLSPGERATLSCRASQSISSSYLAWYQQKPGQAPRLLIYGASIRATGIPDRFSGSGSGTDFTLTISRLEPEDFAVYFCQQYGSSPLTFGGGTKVEIK

MD45- EIVLTQSPGTLSLSPGERATLSCRASQSVNSSYLAWYQQKPGQAPRLLIYGASNRATGIPDRFSGSGSGTDFTLTISRLEPEDFAVYYCQQYGVSPEIIFGQGTRLEIK

MD67- EIVLTQSPGTLSLSPGERATLPCRASQSVSSSYLAWYQQKPGQAPRLLIYGASSRATGIPDRFSGSGSGTDFTLTISRLEPEDFAVYYCQQFGSSPLTFGGGTKVEIK

MD62- AIRMTQSPSSVSASVGDRVTITCRASQDIGSWLGWYQQKPGKAPKLLIYAASSLAGGVPSRFSGSGSGTDFTLTISSLQPEDFATYYCQQANSFPLTFGGGTKLEIK

MD47- NFMLTQPPSVSVAPGKTARITCGGNNIGSKSVHWYQQKPGQAPVLVIYYDSDRPSGIPERFSGSNSGNTATLTISRVEAGDEADYYCQVWDSSSHHHVVFGGGTKGTVL

Supplementary Figure 1. Amino acids sequences of the anti-SARS-CoV-2 antibodies variable regions. Upper panel: Heavy chain variable domains. Lower panel: Light chain variable domains.

<u>**Supplementary Figure 2**</u>

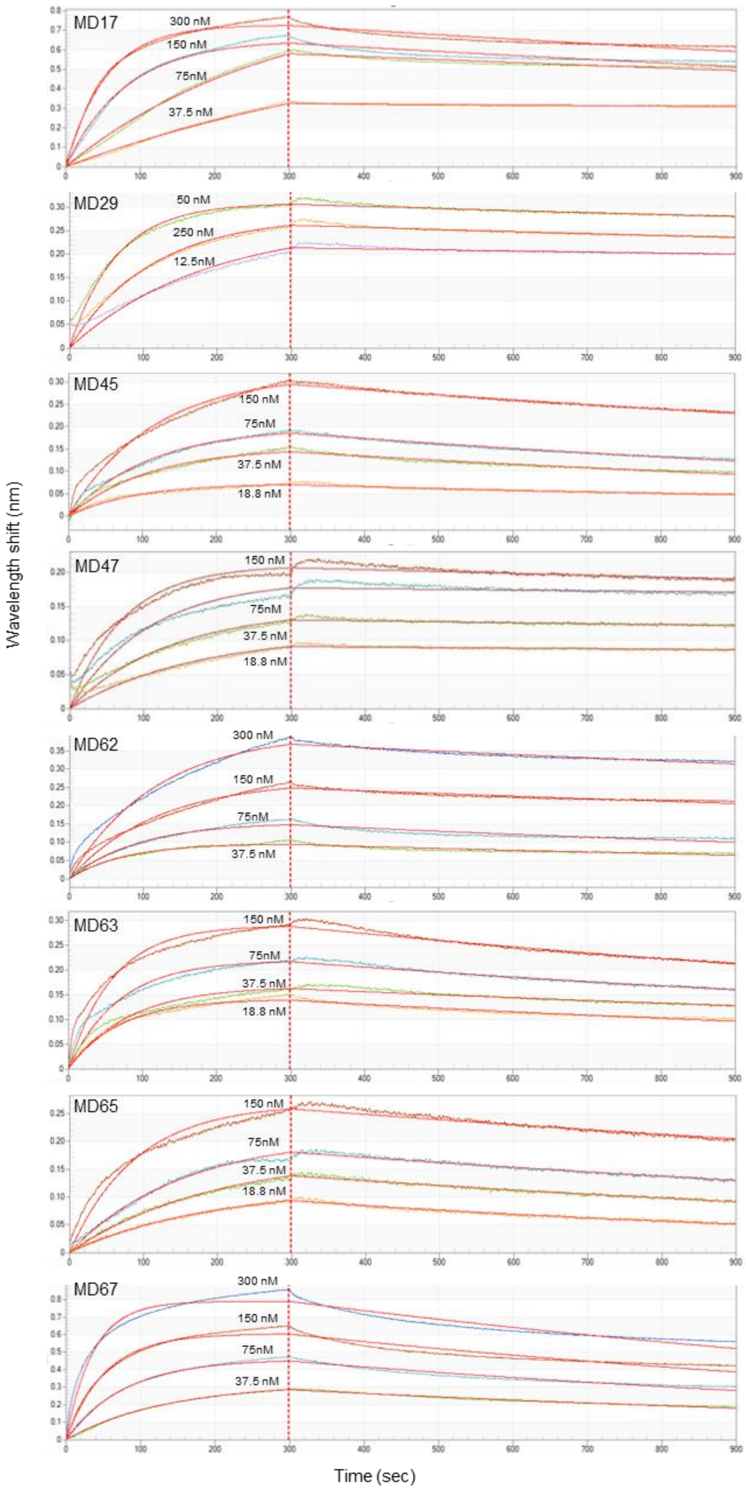

Supplementary Figure 2. **Affinity of antibodies to RBD determined by BLI (Biolayer interferometry) analysis.** Streptavidin-coated biosensors were loaded with biotinylated scFv-Fc antibody and reacted for 300 seconds with the indicated concentrations of monomeric RBD (association phase) and then transferred to buffer-containing wells for another 600 seconds (dissociation phase). Sensorgrams (after subtraction of parallel measurements from unloaded biosensors) were fitted with a 1:1 binding model (red curves) using the Octet data analysis software 8.1.

Supplementary Figure 3

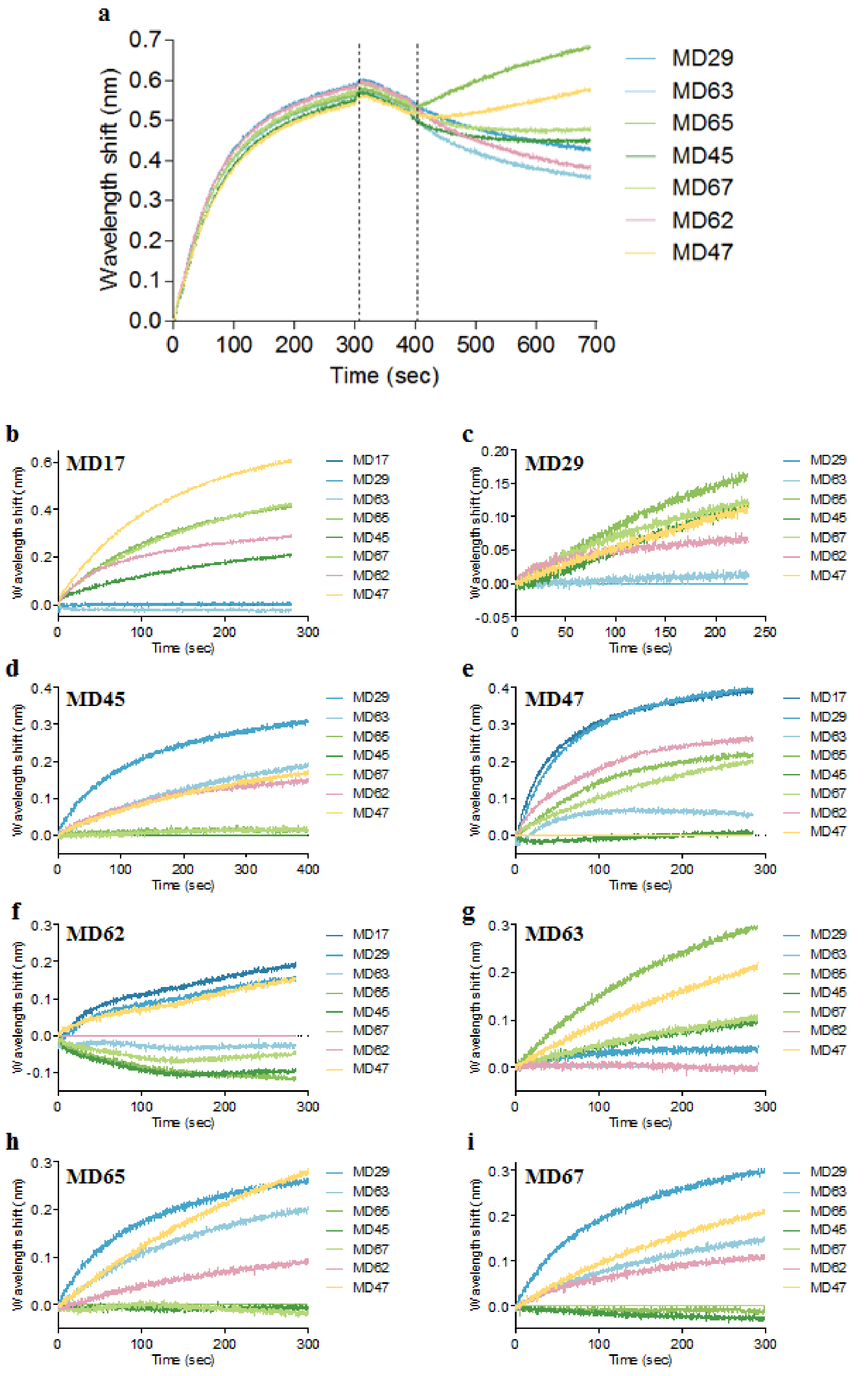

Supplementary Figure 3. Epitope binning of antibodies determined by BLI analysis. (a) Streptavidin-coated biosensors were loaded with biotinylated MD63 antibody and reacted for 300 seconds with the monomeric RBD, washed and then incubated with the indicated antibodies for another 300 seconds. (b-i) The last step is presented for each indicated streptavidin-coated biosensors. In each set of experiments, the background signal was obtained from a parallel sensor incubated with the homologous antibody and sensograms are presented after subtraction of the background signal.

Supplementary Figure 4

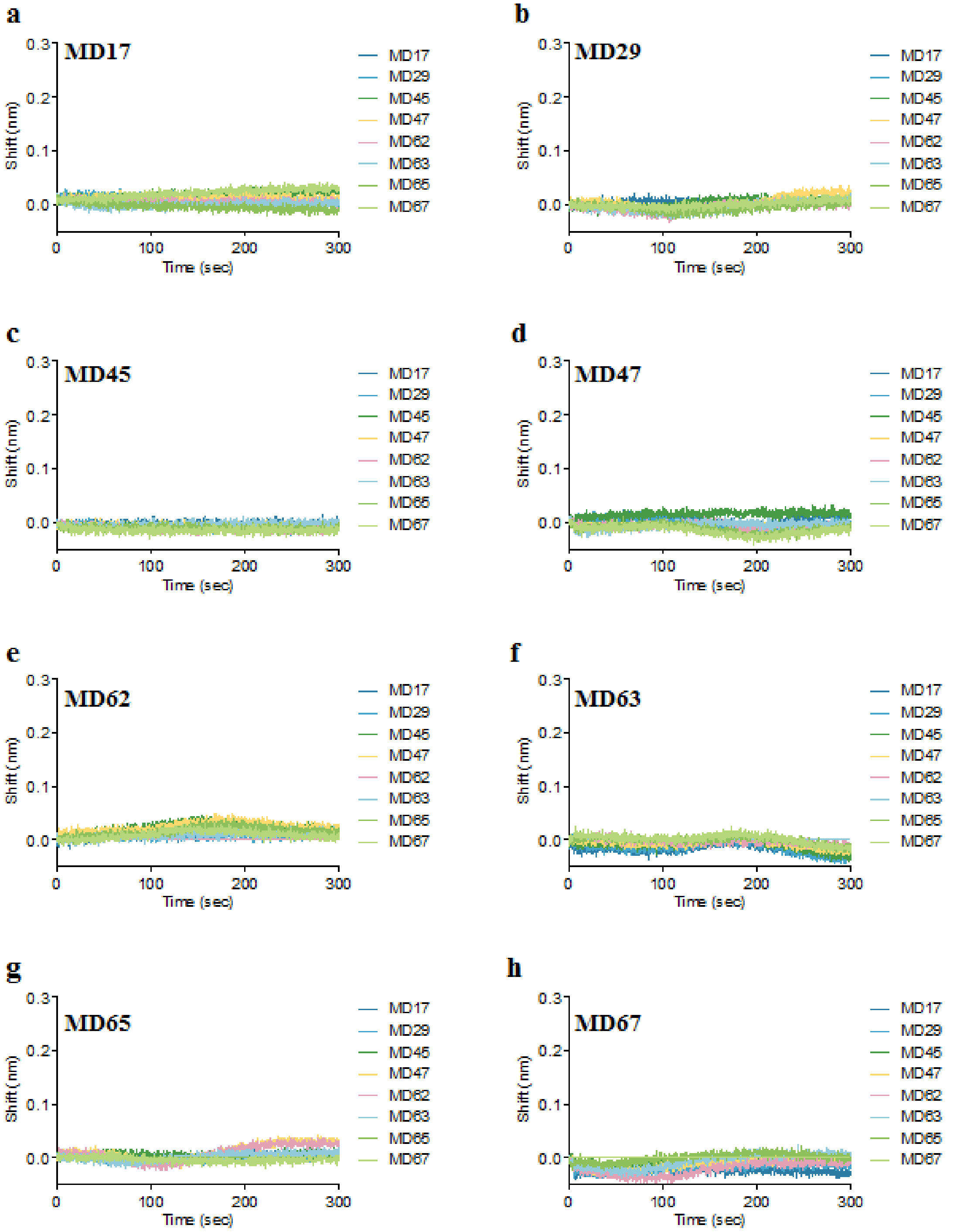

Supplementary Figure 4. **Background control of the BLI epitope binning analysis.** (a-h) Streptavidin-coated biosensors were loaded with the indicated biotinylated antibodies and reacted with the set of antibodies for 300 seconds. The background signal was obtained from a parallel sensor incubated with the homologous antibody and sensograms are presented after subtraction of the background signal.

Supplementary Figure 5

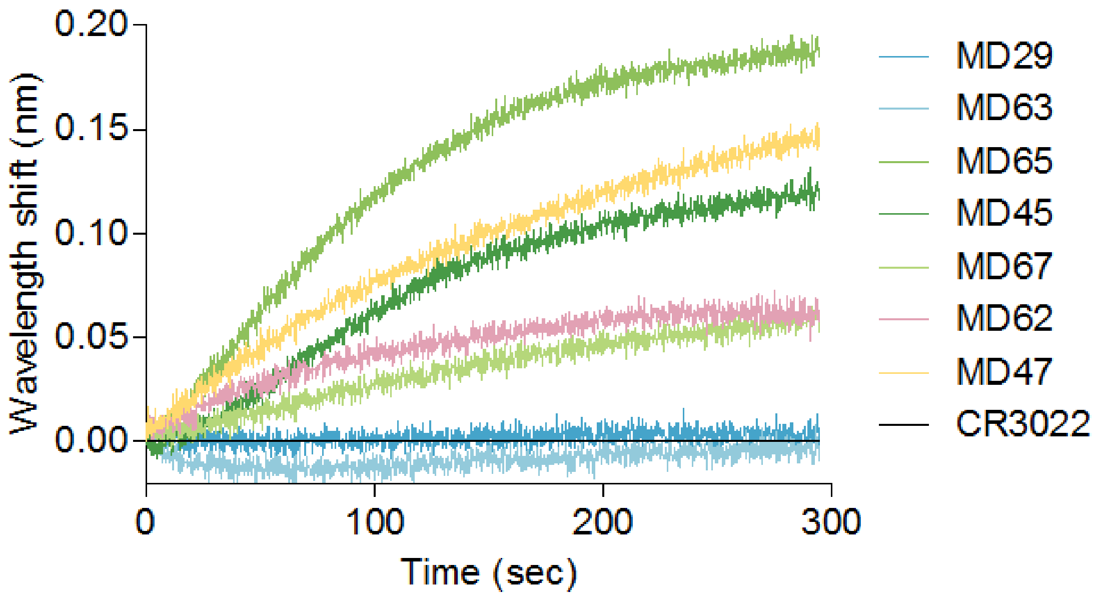

Supplementary Figure 5. BLI epitope binning of selected novel antibodies against antibody CR3022. Streptavidin-coated biosensors were loaded with biotinylated CR3022 antibody and reacted for 300 seconds with monomeric RBD, washed and then incubated with the indicated antibodies for another 300 seconds. The last step is presented for each indicated streptavidin-coated biosensors. Time 0 represents the binding to the CR3022-RBD complex. The background signal was obtained from a parallel sensor incubated with the CR3022 antibody and sensograms are presented after subtraction of the background signal.

Supplementary Figure 6

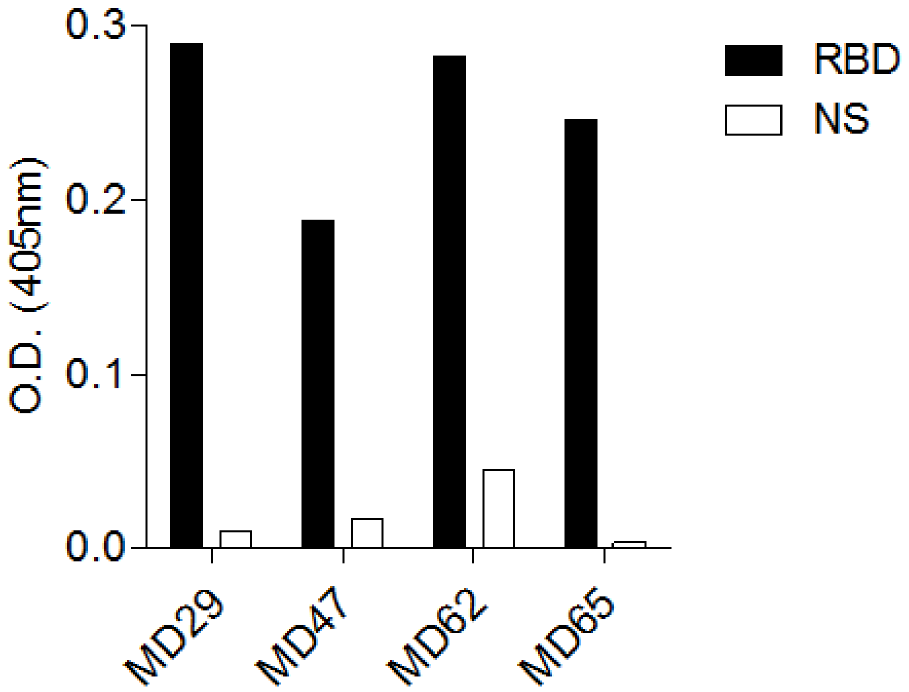

Supplementary Figure 6. **Binding of antibodies to non-glycosylated RBD determined by ELISA.** Microtiter plates were coated with streptavidind, blocked, incubated with recombinant biotinylated RBD that was expressed in *E.coli* and then the indicated antibodies were added to each well. Assay control (NS) was performed in the absence of biotinylated RBD.

<u>**Supplementary Figure 7**</u>

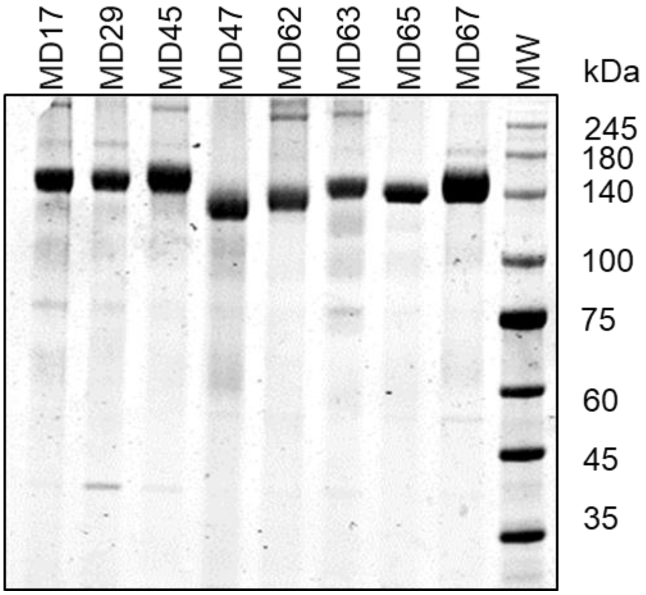

Supplementary Figure 7. **Representative SDS-PAGE analysis of purified scFv-Fc Abs.** The indicated antibodies were expressed in CHO cells, purified on HiTrap Protein-A column and analyzed by SDS-PAGE. 5-7 µg of each Ab boiled at 100°C for 5 min in sample buffer (Laemmli SB; BIORAD, USA) were loaded on each lane. Electrophoresis was performed in 1.5 mm thick NuPAGE^{TM} (4-12% Bis-Tris; Invitrogen, USA), run at 80V for 15 min and at 130 V for additional 60 min. The gel was stained with InstantBlue Coomassie (Expedeon, UK). PM2700 MW (Smobio, Taiwan) protein size markers, are indicated. Identical results were obtained for each of the purified antibodies by at least 3 independent SDS-PAGE analyses.

Supplementary Table

Supplementary Table 1. Primer set for human phage display (PD) library construction

**Primer name**	**Primer Sequence**
**Heavy chain variable region amplification primers**	
Hu -VH-PD_For1	ctttctatgcggcccagccggccatggcc CAGGTBCAGCTKGTRCARTCTGG
Hu -VH-PD_For2	ctttctatgcggcccagccggccatggcc CARATGCAGCTGGTGCAGTCTGG
Hu -VH-PD_For3	ctttctatgcggcccagccggccatggcc GARGTSCAGCTGGTRCAGTCTGG
Hu -VH-PD_For4	ctttctatgcggcccagccggccatggcc CAGATCACCTTGAAGGAGTCTGG
Hu -VH-PD_For5	ctttctatgcggcccagccggccatggcc CAGGTCACCTTGAGGGAGTCTGG
Hu -VH-PD_For6	ctttctatgcggcccagccggccatggcc CAGGTCACCTTGAAGGAGTCTGG
Hu -VH-PD_For7	ctttctatgcggcccagccggccatggcc GARGTRCARCTGGTGGAGTCYGG
Hu -VH-PD_For8	ctttctatgcggcccagccggccatggcc CAGGTGCAGCTGGTGGAGTCTGG
Hu -VH-PD_For9	Ctttctatgcggcccagccggccatggcc GAGGTGCAGCTGTTGGAGTCTGG
Hu -VH-PD_For10	Ctttctatgcggcccagccggccatggcc GAGGTGCAGCTGGTGGAGWCTG
Hu -VH-PD_For11	Ctttctatgcggcccagccggccatggcc CAGGTGCARCTGCAGGAGTCGGG
Hu -VH-PD_For12	Ctttctatgcggcccagccggccatggcc CAGCTGCAGCTGCAGGAGTCSGG
Hu -VH-PD_For13	Ctttctatgcggcccagccggccatggcc CAGGTACAGCTGCAGCAGTCAGG
Hu -JH-PD_Rev1	accaccaccaccggatcctcctcctcctgctgagcc TGAGGAGACRGTGACCAGGG
Hu -JH-PD_Rev2	accaccaccaccggatcctcctcctcctgctgagcc TGAAGAGACGGTGACCATTGTCC
Hu -JH-PD_Rev3	accaccaccaccggatcctcctcctcctgctgagcc TGAGGAGACGGTGACCGTGGTCC
**Kappa chain variable region amplification primers**	
Hu-Vk-PD_For1	ggatccggtggtggtggttctggcggcggcggctcc RHCATCYRGWTGACCCAGTC
Hu-Vk-PD_For2	ggatccggtggtggtggttctggcggcggcggctcc GAYRTYGTGATGACYCAGWC
Hu-Vk-PD_For3	ggatccggtggtggtggttctggcggcggcggctcc GAAATWGTRWTGACRCAGTC
Hu-Vk-PD_For4	ggatccggtggtggtggttctggcggcggcggctcc GAAACGACACTCACGCAGTC
Hu-Vk-PD_For5	ggatccggtggtggtggttctggcggcggcggctcc GAWRTTGTGMTGACWCAGTC
Hu-JK-PD_Rev1	gataccggtgtatttgcgccacctgcggccgc TTTGATHTCCACYTTGGTCC
Hu-JK-PD_Rev2	gataccggtgtatttgcgccacctgcggccgc TTTGATCTCCAGCTTGGTCC
Hu-JK PD_Rev3	gataccggtgtatttgcgccacctgcggccgc TTTAATCTCCAGTCGTGTCC
**Lambda chain variable region amplification primers**	
Hu-VL-PD _For1	ggatccggtggtggtggttctggcggcggcggctcc CAGTCTGTSBTGACKCAGCC
Hu-VL-PD _For2	ggatccggtggtggtggttctggcggcggcggctcc CAGTCTGCCCTGACTCAGCC
Hu-VL-PD _For3	ggatccggtggtggtggttctggcggcggcggctcc TCYTMTGWGCTGACWCAGCC
Hu-VL-PD _For4	ggatccggtggtggtggttctggcggcggcggctcc TCCTATGAGCTGAYHCAGSWVC
Hu-VL-PD _For5	ggatccggtggtggtggttctggcggcggcggctcc CAGSYTGTGCTGACTCAAYC
Hu-VL-PD _For6	ggatccggtggtggtggttctggcggcggcggctcc AATTTTATGCTGACTCAGCC
Hu-VL-PD _For7	ggatccggtggtggtggttctggcggcggcggctcc CAGRCTGTGGTGACYCAGG
Hu-VL-PD _For8	ggatccggtggtggtggttctggcggcggcggctcc CWGSCWGKGCTGACTCAGCC
Hu-JL-PD_Rev1	gataccggtgtatttgcgccacctgcggccgc TAGGACGGTSACCTTSGTCCC
Hu-JL-PD_Rev2	gataccggtgtatttgcgccacctgcggccgc TAGGACGATCAGCTGGGTTCC
Hu-JL-PD_Rev3	gataccggtgtatttgcgccacctgcggccgc TAGGACGGTCAGCTCSGTCCC
Hu-JL-PD_Rev4	gataccggtgtatttgcgccacctgcggccgc TAGGACGGTCASCTKGGTKCC
**Single chain assembly primers**	
ASS1_For	CTTTCTATGCGGCCCAGC
ASS1_Rev	GATACCGGTGTATTTGCGCC
**Single chain_pCC16 amplification primers**	
TAB-RI-For	CCATGATTACGCCAAGCTTTGGAGCC
CBD-As-Rev	GAATTCAACCTTCAAATTGCC

All PCR reactions contained Advantage 2 DNA polymerase mix and reaction buffer (Clontech, USA), 10 mM dNTPs (Promega, USA) and 0.5 µM of each primer [Desalted oligonucleotides (Sigma, Israel)] in a total of 25 µl volume.
